# Oxygen-limited thermal tolerance is seen in a plastron-breathing insect and can be induced in a bimodal gas exchanger

**DOI:** 10.1242/jeb.119560

**Published:** 2015-07-01

**Authors:** Wilco C. E. P. Verberk, David T. Bilton

**Affiliations:** 1 Department of Animal Ecology and Ecophysiology, Institute for Water and Wetland Research, Radboud University, Toernooiveld 1, Nijmegen 6525 ED, The Netherlands; 2 Marine Biology and Ecology Research Centre, School of Marine Science and Engineering, University of Plymouth, Davy Building, Drake Circus, Plymouth PL4 8AA, UK

**Keywords:** Global warming, Heat tolerance, Hypoxia, Multi stressor, OCLTT, Respiration physiology

## Abstract

Thermal tolerance has been hypothesized to result from a mismatch between oxygen supply and demand. However, the generality of this hypothesis has been challenged by studies on various animal groups, including air-breathing adult insects. Recently, comparisons across taxa have suggested that differences in gas exchange mechanisms could reconcile the discrepancies found in previous studies. Here, we test this suggestion by comparing the behaviour of related insect taxa with different gas exchange mechanisms, with and without access to air. We demonstrate oxygen-limited thermal tolerance in air-breathing adults of the plastron-exchanging water bug *Aphelocheirus aestivalis*. *Ilyocoris cimicoides*, a related, bimodal gas exchanger, did not exhibit such oxygen-limited thermal tolerance and relied increasingly on aerial gas exchange with warming. Intriguingly, however, when denied access to air, oxygen-limited thermal tolerance could also be induced in this species. Patterns in oxygen-limited thermal tolerance were found to be consistent across life-history stages in these insects, with nymphs employing the same gas exchange mechanisms as adults. These results advance our understanding of oxygen limitation at high temperatures; differences in the degree of respiratory control appear to modulate the importance of oxygen in setting tolerance limits.

## Abstract (precis)

**Summary:** Oxygen does not appear to universally limit thermal tolerance, but instead, oxygen-limited thermal tolerance is context dependent, being related to a species’ capacity to regulate oxygen consumption.

## INTRODUCTION

Of the many effects of temperature on the physiology of ectotherm animals, it has been argued that thermal limits are set by oxygen limitation at the level of the whole organism (Winterstein, 1905; Brett, 1971; Pörtner, 2006, 2010), since with increasing temperatures there is a progressive mismatch between oxygen supply and demand (see Verberk et al., 2011). The resulting oxygen deficiency initially causes animal performance to decline, and – at more extreme temperatures – aerobic metabolism can no longer be maintained, resulting in heat coma and death due to a lack of oxygen (Pörtner, 2010). This oxygen limitation at the whole-organism level sets in before other physiological systems become thermally impaired (Pörtner, 2006). Oxygen limitation is thus seen as a mechanism that integrates performance across physiological systems, something that can assist our understanding and prediction of thermal effects on growth, reproduction and, ultimately, survival.

Perhaps because of the inherent attractiveness of such a general principle, the hypothesis has become very popular, but has also attracted considerable criticism (e.g. Clark et al., 2013; Norin et al., 2014; Ern et al., 2014; Wang et al., 2014). The prediction that thermal tolerance limits may depend on oxygen delivery has been supported in a number of species (e.g. Frederich and Pörtner, 2000; Mark et al., 2002; Davenport and Davenport, 2007). However, the generality of this mechanism has been perhaps most strongly challenged by studies on terrestrial insects, many of which provide little or no evidence for oxygen-limited thermal tolerance (Klok et al., 2004; Stevens et al., 2010; Molich et al., 2012; Neven et al*.*, 2014). This contrasts with the situation in a number of aquatic insects, whose heat tolerance is apparently much more strongly affected by ambient oxygen availability (Whitney, 1939; Verberk and Calosi, 2012; Verberk et al., 2013a; Verberk and Bilton, 2013).

**Fig. 1.**
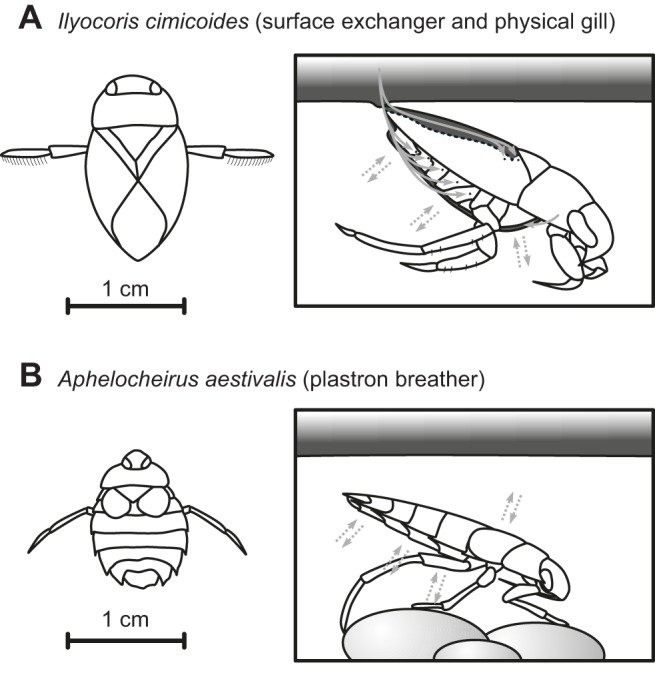
**Overview of gas exchange in the bimodal breather *Ilyocoris cimicoides* and the plastron breather *Aphelocheirus aestivalis*.** Figures are based on Thorpe, 1950 and Popham, 1960. The bimodal breather *I. cimicoides* surfaces periodically to replenish the air that is stored under its hemi-elytra and that is carried on the ventral side. Air is inhaled (inward arrows) via the posterior thoracic spiracles (placed dorsally) and the abdominal spiracles (placed ventrally, except for the first abdominal spiracles) and exhaled (outward arrow) by the anterior thoracic spiracles (placed ventrally). While submerged, the air stored on the ventral side functions as a physical gill (dashed arrows). In contrast, the plastron breather *A. aestivalis*, does not surface and relies solely on diffusion of oxygen from the water. A pile of small hairs covers much of its cuticle and thus a very thin air film is captured and prevented from collapsing. The tracheal spiracles are in open contact with this air film and as it resists changes in volume, oxygen consumed from the air film is renewed by inward oxygen diffusion (dashed arrows). *A. aestivalis* is smaller, more flattened and more rounded, giving it a large surface area to volume ratio, which facilitates oxygen diffusion.

The oxygen requirements of animals and their capacity to deliver oxygen are frequently matched. Fluctuations in both the oxygen requirements of an animal and the environmental availability of oxygen require animals to regulate oxygen delivery in order to match supply to demand. This is important because both a shortage and an excess of oxygen are problematic, leading to asphyxiation and poisoning, respectively. This ability of an organism to regulate oxygen uptake to balance the risks of toxicity and asphyxiation, or its respiratory control, is inherently different between species performing gas exchange in either water or air (Verberk and Atkinson, 2013). In this context, the better respiratory control afforded by breathing air may have driven the evolutionary colonization of land by decapods (see e.g. Giomi et al., 2014). A recent comparative study employing species from different insect orders has suggested that interspecific differences in ability to regulate gas exchange (i.e. the degree of respiratory control) could reconcile these apparently contrasting results between aquatic and terrestrial insects in the degree to which their heat tolerance is set by oxygen limitation (Verberk and Bilton, 2013). However, the eight species investigated in that study differ in more than their mode of respiration, meaning that the contrasting results do not necessarily arise solely from differences in respiratory control, but could also result from other biological differences. Moreover, some of the reported differences in thermal tolerance between aquatic (mostly juvenile stages) and terrestrial insects (mostly adult stages) could also be due to ontogenetic differences in thermal physiology (see e.g. Bowler and Terblanche, 2008; Voorhees and Bradley, 2012) and thus unrelated to respiratory capacity. Here, we compare respiration and thermal tolerance in detail for both juveniles and adults in each of two species of aquatic bugs, one of which is a plastron breather, *Aphelocheirus aestivalis* (Fabricius 1794), and the other a bimodal gas exchanger that is capable of extracting oxygen from air and water, *Ilyocoris cimicoides* (Linnaeus 1758) (see Fig. 1). Moreover, we experimentally manipulate the degree of respiratory control in adults by studying their responses with and without access to air (see below). Such intraspecific comparisons allow more direct testing of the hypothesis that differences in respiratory control govern the extent to which thermal tolerance limits depend on oxygen availability.

Respiration is much more challenging in water compared with air, owing to the lower solubility and diffusivity of oxygen in water, whilst the higher density and viscosity of water increase the cost of breathing (Dejours, 1981; Verberk and Atkinson, 2013). With this in mind, we can derive several hypotheses regarding the responses of the study organisms based on their mode of respiration. With respect to oxygen consumption, we expect that of the plastron breather to be lower than that of the bimodal breather, owing to the greater challenge of breathing under water than in air. Furthermore, differences in oxygen consumption between both species are expected to become larger at higher temperatures, with the bimodal breather relying more and more on aerial gas exchange. With respect to temperature, we expect the extent of oxygen-limited thermal tolerance to be a function of the capacity of an animal to regulate oxygen uptake. If the plastron breather is indeed worse at regulating oxygen uptake than the bimodal breather, we expect its heat tolerance to be more oxygen dependent, with a greater impact of both hypoxia and hyperoxia. Furthermore, juveniles and adults of a given species should perform similarly in heating trials if their gas exchange mechanisms are comparable. Finally, and most crucially, we predict that the heat tolerance of the bimodal breather becomes much more oxygen dependent when it is denied access to air, since restricting such access would reduce its respiratory control.

## RESULTS

**Fig. 2.**
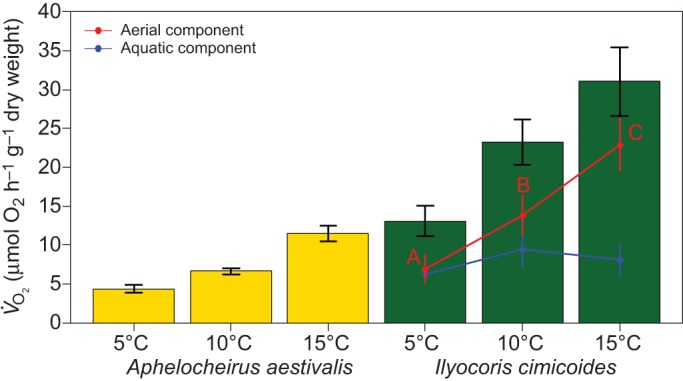
**Differences in mass-specific oxygen consumption between *Aphelocheirus aestivalis* and *Ilyocoris cimicoides* at different temperatures.** For the bimodal breather *I. cimicoides*, aerial respiration (red line) and aquatic respiration (blue line) are indicated separately. Error bars indicate s.e.m. and different letters indicate significant differences in aerial respiration (*P*<0.05) between temperatures (note that aquatic respiration did not differ between temperatures for *I. cimicoides*).

Metabolism increased with both temperature (Fig. 2) and body size, and for a given body size, the bimodal breather *I. cimicoides* consumed more oxygen than the plastron breather *A. aestivalis* (Table 1). Also, in warmer conditions, *I. cimicoides* increased its oxygen consumption more than *A. aestivalis* (Fig. 2; GLM species×temperature, *F*_1,74_=8.65; *P*=0.0044). In warmer conditions, *I. cimicoides* relied increasingly on aerial gas exchange; proportionally more oxygen was consumed from air with increasing temperatures (Fig. 2; linear regression β=0.015; *F*_1,33_=20.25; *P*<0.001), whereas, oxygen consumption from water did not change (linear regression β=0.0019; *F*_1,33_=0.575; *P*=0.454). Thus, there are clear differences between both species in their rates of oxygen consumption and the thermal dependence of this; *I. cimicoides* consumes more oxygen and can increase its consumption more with increasing temperatures by relying on more frequent aerial gas exchange.

**Table 1.**
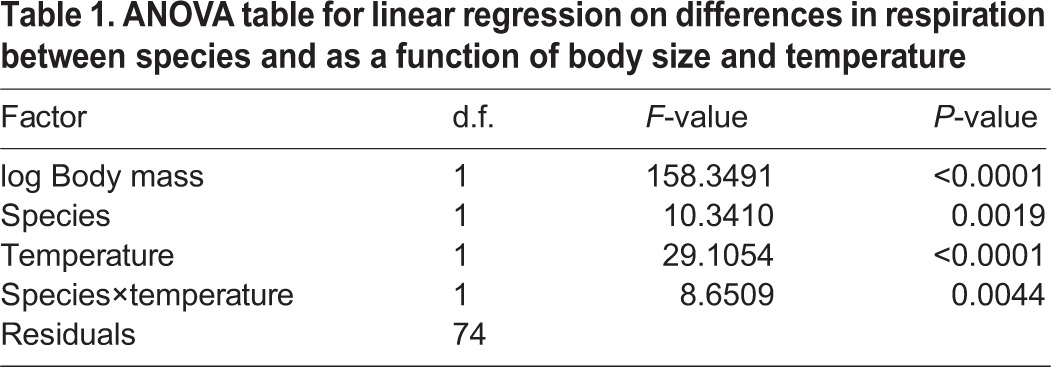
**ANOVA table for linear regression on differences in respiration between species and as a function of body size and temperature**

**Fig. 3.**
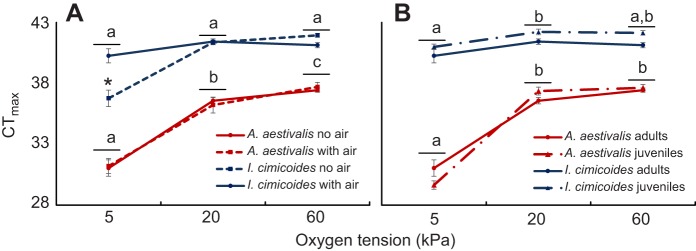
**Differences in heat tolerance between *Aphelocheirus aestivalis* and *Ilyocoris cimicoides* at different temperatures.** Differences for adults with and without access to air (A) are shown separately from differences between adults and juveniles (B). For ease of comparison, the line for adults is repeated in both panels. Error bars indicate s.e.m.

Heat tolerance was also different between the two species, with *I. cimicoides* showing higher heat tolerance than *A. aestivalis* at each oxygen tension tested (Fig. 3; GLM species, *F*_1,128_=270.78, *P*<0.001). Oxygen affected heat tolerance of the two species to differing degrees (GLM species×oxygen interaction *F*_1,128_=17.25, *P*<0.001), having a much more pronounced effect in *A. aestivalis*.

In adults of *I. cimicoides*, hypoxia did not significantly reduce heat tolerance (Fig. 3A; *t*=−1.93; *P*=0.060) so long as animals had access to air. However, when *I. cimicoides* was deprived of air, heat tolerance was reduced significantly under hypoxia by 4.6°C (*t*=−3.84; *P*<0.001). In contrast, adults of *A. aestivalis* showed both reduced heat tolerance under hypoxia and improved heat tolerance under hyperoxia (Fig. 3A). Provision of adult *A. aestivalis* with access to air did not improve heat tolerance under hypoxia (Fig. 3A), because *A. aestivalis* never attempted to surface in any experimental treatment.

Juveniles and adults did not differ in heat tolerance across the two species (Fig. 3B; GLM stage, *F*_1,99_=1.419, *P*=0.237). Nevertheless, including juveniles in the analyses did affect the statistical significance compared with the analyses on adults only. There was a modest overall effect of hypoxia on heat tolerance in *I. cimicoides* (*t*=−2.490, *P*=0.0144), whilst the effect of hyperoxia in *A. aestivalis* was marginally non-significant (*t*=1.758, *P*=0.082). Nevertheless, in general, the results were highly similar to those for only adults (Fig. 3B), showing much stronger effects of oxygen in the plastron breathing *A. aestivalis*.

## DISCUSSION

A mechanistic understanding is essential to predict the impacts of environmental stressors, and comparisons of species attributes or traits offer a promising approach to unravel such mechanisms (Huey et al., 2012; Chown, 2012; Verberk et al., 2013b). Temperature is a primary driver of species distribution (e.g. Root et al*.*, 2003; Calosi et al*.*, 2010; Sunday et al*.*, 2011) and the thermal tolerance of a species has been argued to be one of the key traits predicting biological responses to a rapidly changing climate (Chown, 2012). Our study supports the view that the extent to which the mechanism of oxygen-limited thermal tolerance applies is context dependent and related to differences in respiratory control. As the critical lethal temperatures reported here will be higher than sublethal limits to growth and reproduction, these cannot be related directly to the habitat conditions in which these bugs live. However, our study provides a clear proof of principle that the extent to which oxygen limits thermal tolerance is a function of the respiratory control of an animal, rather than oxygen-limited thermal tolerance being universally applicable. Such a result may offer a way forward in the current debate between proponents and opponents of the generality of this conceptual framework (e.g. Clark et al., 2013; Pörtner, 2014).

Differences in respiratory control arise, in part, from the respiratory medium. Underwater gas exchange has higher ventilation costs than aerial exchange, because water is more dense and viscous than air; a problem that is further compounded by lower oxygen content and much lower diffusion rates in liquid media (Dejours, 1981; Verberk and Atkinson, 2013). This difficulty of breathing in water rather than air is also exemplified by the finding of Jones (1961) who observed that pulmonate snails increased their reliance on aerial oxygen under aquatic hypoxia. Likewise, we found that the bimodal gas exchanger *I. cimicoides* increasingly relied on aerial gas exchange under warmer conditions (Fig. 2). The plastron breather *A. aestivalis* did not show such a response, however, and was less efficient in obtaining oxygen at higher temperatures. This latter species also showed clear evidence for oxygen-limited thermal tolerance, with heat tolerance being greatly compromised under hypoxic conditions, whilst hyperoxia somewhat improved heat tolerance (Fig. 3).

Finding that hypoxia depresses CT_max_ does not in itself imply that oxygen also limits thermal tolerance under normoxia (e.g. Molich et al., 2012; Neven et al., 2014). Therefore, elevated CT_max_ under hyperoxia is usually seen to constitute a stronger test of the hypothesis that oxygen limits thermal tolerance. Here, it has to be borne in mind that we only assessed short-term responses to acute heating, whereby animals did not have to actively perform, but rather meet minimum oxygen requirements for survival. Studies have shown that oxygen can become more readily limiting when animals have oxygen requirements beyond standard metabolism (because of activity, feeding, growing etc.). For example, the dragonfly *Erythemis simplicicollis* was found to be oxygen limited during flight, even in normoxia (Harrison and Lighton, 1998). Thus, when animals have to actively perform they may become oxygen limited, and the likelihood of this happening could be approximated by the decrease in CTmax under hypoxia (assuming that a more challenging oxygen uptake under hypoxia is equivalent to a greater requirement of oxygen in active animals under normoxia). From this perspective, the bimodal gas exchanger *I. cimicoides* seems unlikely to be oxygen limited when it has access to air. In a similar manner, the increased respiratory control afforded by aerial gas exchange appears to increase heat tolerance in some intertidal decapods (Giomi et al., 2014; Fusi et al., 2015). If not set by oxygen, thermal limits can arise as a result of other processes, such as the loss of protein function due to denaturation, loss of membrane stability and neuronal dysfunction (Somero, 1995; Feder and Hofmann, 1999; Klok et al., 2004; Verberk et al., 2013a). If the critical temperatures for these other processes are only slightly higher than the temperature at which oxygen becomes limiting, then hyperoxia will only have minor effects, alleviating constraints on oxygen delivery, but with the next process in line breaking down at a temperature very close to the CT_max_ observed under normoxia. From this perspective even a slight increase in thermal tolerance under hyperoxia observed for the plastron breather could, together with the large decline in thermal tolerance under hypoxia, indicate that it may become oxygen limited even under normoxic conditions. Thus, in addition to tegument- and gill-breathing aquatic insects, we can add that plastron-breathing insects are also likely to be vulnerable to oxygen limitation. This is the second example of an insect in which hyperoxia results in improved heat tolerance (see Verberk and Bilton, 2011 for the first case), and the first case for insect species with an open tracheal system. Contrary to the conclusions of Klok et al. (2004), it seems that adult insects with open tracheal systems can indeed suffer from oxygen-limited thermal tolerance, at least if, like plastron breathers, they have limited respiratory control. In short, animals that have difficulty in regulating oxygen consumption are more likely to become oxygen limited, irrespective of whether they are tracheates or otherwise.

Previous comparisons between plastron breathers and aerial gas exchangers have shown the former to be especially vulnerable to warming and hypoxia, presumably because of their poor respiratory control (Verberk and Bilton, 2013). Here, this pattern is confirmed and extended to hyperoxia (Fig. 3). More importantly, our experimental manipulations enable us to isolate the effect of respiratory control within a single species. By denying the bimodal breather access to air, we reduce the respiratory control of *I. cimicoides*, and this resulted in a clear reduction of heat tolerance under hypoxia by as much as 4.6°C. This makes it very likely that respiratory control is the driving factor underlying the extent to which oxygen limits thermal tolerance, explaining the differences in support across a range of species (Verberk and Bilton, 2013). From our results, it is also unlikely that ontogenetic differences in thermal biology play a large role. Differences in thermal tolerance between life stages could be inherent, irrespective of differences in their gas exchange mechanism, arising because life stages may differ in their ability to thermoregulate (e.g. Bowler and Terblanche, 2008; Voorhees and Bradley, 2012). In holometabolous insects, larvae frequently differ from adults in their gas-exchange mechanisms, making it difficult to disentangle the potential effects of ontogenetic shifts in thermal physiology and the effect of respiratory control. In the hemimetabolous bugs studied, comparing juveniles and adults and keeping the gas exchange mechanism constant, we showed very similar levels of heat tolerance for both life stages, suggesting that differences between instars may not result from ontogenetic differences in their thermal physiology (Verberk and Bilton, 2011). A similar heat tolerance across life stages also implies that the capacity to increase oxygen consumption does not differ markedly with body size in these insects. For terrestrial insects, critical *P*_O_2__ (the oxygen tension below which metabolic rate is depressed) was found to be indeed independent of size (Greenlee et al., 2007; Harrison et al., 2014) as a result of compensatory changes in tracheal conductance (e.g. Loudon, 1989). Our results suggest that across the 2- to 3-fold size range, the two hemipterans studied here could maintain capacity for oxygen delivery with increasing size. In tegument-breathing stonefly larvae, a modest but significant effect was found for body size on heat tolerance (Verberk et al., 2013a). This could be related to the larger 8-fold size range in the stonefly nymphs and their poor respiratory control associated with tegument breathing.

In conclusion, our study significantly advances our understanding of oxygen limitation at warm conditions, demonstrating that respiratory control dictates the importance of oxygen in setting heat tolerance limits. As differences in respiratory control will often be tightly linked to their respiratory medium (air versus water), this finding reconciles the contrasting results on thermal tolerance limits previously reported for terrestrial and aquatic insects. Based on our results, we predict that the vulnerability of species to the interactive effects of warming and hypoxia will be dictated by their respiratory control. Thus, within water breathers, thermal generalists are expected to have excellent respiratory control (Ern et al., 2014). Conversely, species with poor respiratory control are predicted to be highly vulnerable. Species in running waters are more likely to fall into this category because their habitat does not require good respiratory control: water flow greatly reduces the ventilation effort needed, while the resultant mixing of air and water ensures consistently high oxygen saturation. Thus, respiratory control is a key trait which must be included in studies that aim to predict biological responses to a rapidly changing climate.

## MATERIALS AND METHODS

### Study species

Animals were collected in SW England. *Aphelocheirus aestivalis* was collected from riffles in the River Torridge, Devon, UK (50.9094, −4.0743), a medium to fast flowing river with a stony bed and little vegetation. In the River Torridge, temperature ranges from 6.2–19.2°C with an average of 10.9°C (data obtained from ongoing monitoring by the UK Environment Agency). *Ilyocoris cimicoides* was collected from South Drain, Somerset Levels, UK (51.1806, −2.8803), a slow flowing, drainage canal with abundant riparian and aquatic macrophytes. In the South Drain, temperature ranges from 10.1–24.6°C with an average of 16.7°C (Dalesman and Rundle, 2010). After collection, animals were maintained in the Marine Biology and Ecology Research Centre in a constant temperature room at 10±1°C in a 12 h:12 h L:D regime. They were fed chironomid larvae *ad libitum* and were kept in aquaria, containing artificial pond water (ASTM, 1980), buffered and diluted to reflect the pH and conductivity of the field site. All nymphs were acclimated for at least 7 days to lab conditions to reduce variability in thermal history (see Terblanche et al., 2007), before respiration trials and trials to assess critical temperatures were undertaken.

The plastron-breathing heteropteran *A. aestivalis* does not need to surface to renew its air supply. Instead, it relies on diffusion of oxygen from the water into the thin film of air that is in contact with its tracheal system via the spiracles (see Fig. 1). A pile of small hairs covers much of the cuticle and prevents the air film from collapsing (Thorpe, 1950). As it resists changes in volume, oxygen consumed from the air film is renewed by inward oxygen diffusion and the animal can maintain respiration indefinitely under normal conditions. In comparison to the other heteropteran *I. cimicoides*, *A. aestivalis* has a larger surface area to volume ratio, facilitating oxygen diffusion (see Fig. 1). Respiration rates are relatively low, which seems to be a general feature of plastron-breathing insects (Kölsch and Krause, 2011). Body weights (mg dry weight) ranged from 5.3–9.3 mg to 11.4–17.3 mg and 14.9–24.7 mg for juveniles, males and females, respectively (measured to the nearest 0.0001 g), with dry weight making up 44.9% of fresh weight.

The other heteropteran investigated, *Ilyocoris cimicoides*, is very similar in morphology and diet. Moreover, recent work on the phylogeny of these hemipterans suggests that the families to which both species belong (Aphelocheiridae and Naucoridae, respectively) are sister taxa (Fig. 3 in Hua et al., 2009), with the Naucoridae being morphologically similar to Aphelocheiridae (Hebsgaard et al., 2004). *Ilyocoris cimicoides* has an air store under its hemi-elytra and it also carries a thin film of air on the ventral side which functions like a physical gill, but unlike *A. aestivalis*, the bubble cannot be maintained indefinitely and the species therefore needs to surface to renew its air stores (Fig. 1). This species is consequently a bimodal breather, using the thin layer of air as a physical gill and surfacing periodically to replenish its air stores. Being hemimetabolous, juveniles of both species employ gas exchange mechanisms identical to those of the adults. Body weights (mg dry weight) ranged from 7.8–21.8 mg to 22.3–36.6 mg and 23.0–54.8 mg for juveniles, males and females, respectively (measured to the nearest 0.0001 g), with dry weight making up 34.9% of fresh weight.

### Respiration measurements

To measure respiration rates in the bimodal breather, *I. cimicoides*, we used cylindrical respiratory chambers (115 mm high, 27 mm inner diameter) with a cone-shaped top (sides started tapering 15 mm from the top). These chambers were fully filled with water before animals were inserted. Immediately after an animal was inserted, we injected 2 ml of air that was saturated with water vapour and temperature equilibrated. The resulting air bubble was held at the conical top of the respiratory chamber (hereafter called air compartment) and proved sufficiently spacious for the animal to perform aerial gas exchange. Animals were given between 1 and 1.5 h to acclimate. Next, oxygen consumption was measured by measuring the oxygen tension in the water and air compartment, using a micro-optodes connected to a Microx TX3 fiber-optic oxygen meter (PreSens instruments, Regensburg, Germany). The micro-optode was situated in the air compartment. Oxygen tensions in the water compartment were measured at the start and end of each respiration trial by carefully inverting the respiratory chamber to displace the air bubble and allow the optode to come into contact with the water. Water was stirred throughout the respiration, using submersible stirrers (Cole-Palmer Instrument Company Ltd., London, UK). A specifically constructed mesh separated the stirring bar from the animal. This also provided animals something to cling on to, thus minimizing activity. Oxygen tensions in the air compartment were measured multiple times and a linear regression line was fitted to calculate oxygen consumption. During the experiment, oxygen could freely diffuse between the air and water compartment (i.e. there was no paraffin barrier added to the water). As the oxygen tension in the air compartment was usually lower than that of the water compartment, such diffusion of oxygen across the air–water interface could underestimate the oxygen consumed from air and overestimate the oxygen consumed from the water. We therefore corrected for this by empirically measuring the diffusive flux over a range of differences on *P*_O_2__ at different temperatures. Corrected and uncorrected values were highly similar (e.g. for aerial consumption, corrected values were on average 7% higher than uncorrected values and highly correlated; *R*^2^=0.996), indicating that the contribution of diffusion of oxygen across the air–water interface was relatively modest in comparison to oxygen consumption by the bugs. Respiration trials lasted between 113 and 182 min, in accordance with the rates of oxygen consumption of the animal, which differed with size and temperature. On average, oxygen saturation dropped to 90% at the end of a respiration trial, but never below 71%. Oxygen consumption was corrected for background respiration, which was assessed by blanks.

To measure respiration rates in the plastron breather *A. aestivalis*, we used cylindrical respiratory chambers without an air compartment and which were of smaller size (50 mm high, 15 mm inner diameter). Respiration trials lasted between 66 and 241 min, in accordance with the rates of oxygen consumption of the animal, which differed with size and temperature. On average, oxygen saturation dropped to 96% at the end of a respiration trial, but never below 82%. To minimize background respiration, respiratory chambers were cleansed with 100% ethanol before use, and thoroughly washed and dried afterwards. Oxygen consumption was corrected for background respiration, which was assessed by blanks. For both species, respiration rates were measured at three temperatures: 5°C, 10°C and 15°C. To this end, respiratory chambers were submerged in water maintained at 5°C, 10°C or 15°C in a Grant R5 water bath with a GP200 pump unit (Grant Instruments Ltd, Cambridge, UK). Volume of the respiratory chambers was determined gravitationally from the difference in weight when empty or completely filled with water.

### Assessing CTmax

In order to assess critical temperatures, we employed the same methods as previously described (Verberk and Bilton, 2011; Verberk and Calosi, 2012). Briefly, individual nymphs were placed in five parallel flow-through chambers (70×70×30 mm; flowrate, 0.016 l s^−1^) and water was supplied to these chambers by gravity from a 25 l header tank after having passed through a tubular counter-current heat exchanger. Water in the header tank was of the same composition as that used to maintain animals and was bubbled with a mixture of 20% O_2_ and 80% N_2_, obtained using a gas-mixing pump (Wösthoff, Bochum, Germany). Individuals were left resting for 1 h at the equilibration temperature of 10°C, after which temperature in the experimental chambers was increased at 0.25°C min^−1^, using a Grant R5 water bath with a GP200 pump unit, connected to the heat exchanger. Temperatures were logged using a HH806AU digital thermometer (Omega Engineering Inc., Stamford, USA).

The critical maximal temperature, CTmax, was defined as loss of all movement. At this temperature, animals no longer showed any body movement or muscular spasms and could be most reliably determined across the different taxa. It is considered a comparable endpoint as animals in this state lose their ability to escape from the conditions that will lead to their death (Lutterschmidt and Hutchison, 1997). At lower temperatures, animals initiated repeated swimming behaviour (interpreted as attempts to escape experimental conditions) and fell upon their backs (loss of equilibrium) and an onset of spasms was observed which eventually prevented the animal from coordinated swimming strokes with the two hind legs.

CTmax was assessed at normoxia, hypoxia and hyperoxia. Different levels of oxygenation were achieved by changing the O_2_–N_2_ gas mixture obtained using the gas-mixing pump. The gas mixture was adjusted 10 min after placing the animals in the small flow-through chambers, to allow for gradual exposure to hypoxic and hyperoxic conditions during the 1 h resting period. To prevent equilibration with the atmosphere, the header tank was sealed using an 18-mm-thick expanded polystyrene sheeting and other openings were sealed with plastic material. During the 1 h resting period, O_2_ levels in the outflow water from the chambers were measured approximately every 15 min, to verify that the O_2_ levels had stabilized to hypoxic (5 kPa), normoxic (20 kPa) and hyperoxic (60 kPa) conditions at the onset of warming. Because some equilibration with the atmosphere could not be prevented, nominal output values from the gas mixer were slightly more extreme (3 kPa for hypoxia and 65 kPa for hyperoxia) in order to achieve the desired O_2_ conditions in the test chambers.

Adults were assessed with and without access to air, while juveniles were assessed with access to air for *I. cimicoides* and without access for *A. aestivalis*. To assess CTmax with the animals having access to air, we used a flow-through chamber with a small head space holding a layer of air. The air in this compartment could be flushed via a narrow opening that was sealed from the atmosphere by the capillary action of water. Using a thin needle connected to the gas-mixing pump, we could flush the head space of the flow-through chamber with different gas mixtures of oxygen and nitrogen. Equilibration of the head space with the atmosphere was prevented by the capillary action of water, which sealed off the opening immediately after the needle was retracted. To ensure that the air and water compartments held the same gas mixture at the onset of the warming, we flushed the head space of the flow-through chamber with air of the appropriate gas mixture, both 10 min after the start of the resting period (when water oxygen content was also adjusted) and at the end (when warming was initiated).

### Data analysis

Fitted residuals for the respiration data deviated slightly from a normal distribution. Visual inspection revealed that the deviance of the residuals increased with higher predicted values (funnel-shaped error distribution). To evaluate the robustness of the model outcome, we therefore also fitted a GLM using a gamma distribution. As the results from both analyses were qualitatively highly similar, we only present those of the simpler linear regression. Moreover, respiration of the same individual was measured at different temperatures. To account for the non-independence of data points from the same individual, we also ran a mixed-effect linear regression using individual as a random factor. Again, this analysis confirmed the results from the simpler linear regression (higher metabolism at higher temperatures, which was stronger for *I. cimicoides*).

Preliminary analysis on heat tolerance revealed differences between both species in their heat tolerance, in how oxygen conditions affected their heat tolerance and in how the experimental setup interacted with this effect of oxygen on heat tolerances (see supplementary material Table S1). As these latter three-way interactions are difficult to interpret, we decided to analyse the effects of the experimental setup (access or no access to air) in two analyses for each species separately (see supplementary material Tables S2 and S3). Also, since we only manipulated access to air for adults we restricted this analysis to adults. To assess whether heat tolerance was consistently different between juveniles and adults, we conducted a third analysis whereby we included data for both species (supplementary material Table S4), including data on juveniles and adults that were assessed at the same experimental conditions (no access to air for the permanently submerged plastron breather and access to air for the bimodal breather).

## Supplementary Material

Supplementary Material
