# Supplementary material for: Oxygen-limited thermal tolerance is seen in a plastron-breathing insect and can be induced in a bimodal gas exchanger
Source: J Exp Biol. 2015 Jul 1;218(13):2083–8. doi: 10.1242/jeb.119560 (PMC4510840; doi:10.1242/jeb.119560)
Supplement: Supplementary Material [file supp_218_13_2083__index.html]

Supplementary Material 

# Oxygen limited thermal tolerance is seen in a plastron breathing insect, and can be induced in a bimodal gas exchanger

## JEB119560 Supplementary Material

- Supplementary Material
