# Supplementary material for: Oxygen-limited thermal tolerance is seen in a plastron-breathing insect and can be induced in a bimodal gas exchanger
Source: J Exp Biol. 2015 Jul 1;218(13):2083–8. doi: 10.1242/jeb.119560 (PMC4510840; doi:10.1242/jeb.119560)
Supplement: Supplementary Material [file supp_jeb.119560_JEB119560supp.pdf]

Table S1. ANOVA table for linear regression on CTmax data for all individuals

```
Response: data$CTmax1

                      Df  Sum Sq Mean Sq  F value    Pr(>F)    
O2                     1  286.55  286.55  69.5651  1.01e-13 ***
Species                1 1115.40 1115.40 270.7805 < 2.2e-16 ***
Expsetup               1   15.03   15.03   3.6481   0.05837 .  
log10(DW)              1    0.28    0.28   0.0674   0.79560    
Stage                  1    7.98    7.98   1.9382   0.16628    
O2:Species             1   71.06   71.06  17.2517  5.94e-05 ***
O2:Expsetup            1   24.09   24.09   5.8477   0.01700 *  
Species:Expsetup       1   15.20   15.20   3.6908   0.05694 .  
O2:Species:Expsetup    1   19.02   19.02   4.6165   0.03355 *  
Residuals            128  527.26    4.12
```

Table S2. Summary of linear regression on CTmax data for *Ilyocoris* adults

```
lm(formula = CTmax1 ~ oxygen + Expsetup + log10(DW) + Expsetup:oxygen, 
    data = subset(ilyo, ilyo$Stage != "juv"))

Residuals:
    Min      1Q  Median      3Q     Max 
-5.0109 -0.4133  0.1690  0.5986  2.5541 

Coefficients:
                         Estimate Std. Error t value Pr(>|t|)    
(Intercept)              47.23300    5.57956   8.465 6.15e-11 ***
oxygen0.05               -1.06163    0.54952  -1.932 0.059541 .  
oxygen0.6                -0.23786    0.55757  -0.427 0.671654    
Expsetupnoair             0.05135    0.70832   0.072 0.942520    
log10(DW)                -2.32237    2.22779  -1.042 0.302648    
oxygen0.05:Expsetupnoair -3.59189    0.93629  -3.836 0.000379 ***
oxygen0.6:Expsetupnoair   0.78553    1.06128   0.740 0.462956    
---
Signif. codes:  0 ‘***’ 0.001 ‘**’ 0.01 ‘*’ 0.05 ‘.’ 0.1 ‘ ’ 1
```

Table S3. Summary of linear regression on CTmax data for *Aphelocheirus* adults

```
lm(formula = CTmax1 ~ O2 + Expsetup + log10(DW) + Stage + O2:Expsetup +
    Expsetup:O2, data = aphe)

Residuals:
    Min      1Q  Median      3Q     Max
-4.9723 -0.6602  0.0136  0.7151  4.4284

Coefficients:
                     Estimate Std. Error t value Pr(>|t|)
(Intercept)           29.1112     4.2081   6.918 2.42e-09 ***
O20.2                  4.9774     0.9341   5.328 1.33e-06 ***
O20.6                  6.4267     0.9416   6.825 3.53e-09 ***
Expsetupnoair         -0.4826     0.7525  -0.641    0.524
log10(DW)              0.9482     1.8867   0.503    0.617
Stagejuv               0.3786     0.9576   0.395    0.694
O20.2:Expsetupnoair    1.0179     1.0413   0.977    0.332
O20.6:Expsetupnoair    0.3254     1.0648   0.306    0.761
---
Signif.
```

Table S4. ANOVA table of linear regression on CTmax data for adults and juveniles of both the permanently submerged plastron breather *Aphelocheirus* (assessed without access to air) and the bimodal breather *Ilyocoris* (assessed with access to air)

```
Response: CTmax1
                Df Sum Sq Mean Sq  F value    Pr(>F)
oxygen           2 340.14  170.07  95.4400 < 2.2e-16 ***
Species          1 978.04  978.04 548.8599 < 2.2e-16 ***
Stage            1   2.53    2.53   1.4186    0.2365
oxygen:Species   2 160.85   80.42  45.1327 1.077e-14 ***
Residuals      100 178.20    1.78
---
Signif. codes:  0 ‘***’ 0.001 ‘**’ 0.01 ‘*’ 0.05 ‘.’ 0.1 ‘ ’ 1
```
